# Supplementary material for: Novel Driver Strength Index highlights important cancer genes in TCGA PanCanAtlas patients
Source: PeerJ. 2022 Aug 11;10:e13860. doi: 10.7717/peerj.13860 (PMC9375969; doi:10.7717/peerj.13860)
Supplement: Supplemental Information 2 [file peerj-10-13860-s002.zip › COHORTS/genes plots/cohorts/2021_8_16_14_9_distribution_cohorts_males_PANCAN.pdf]

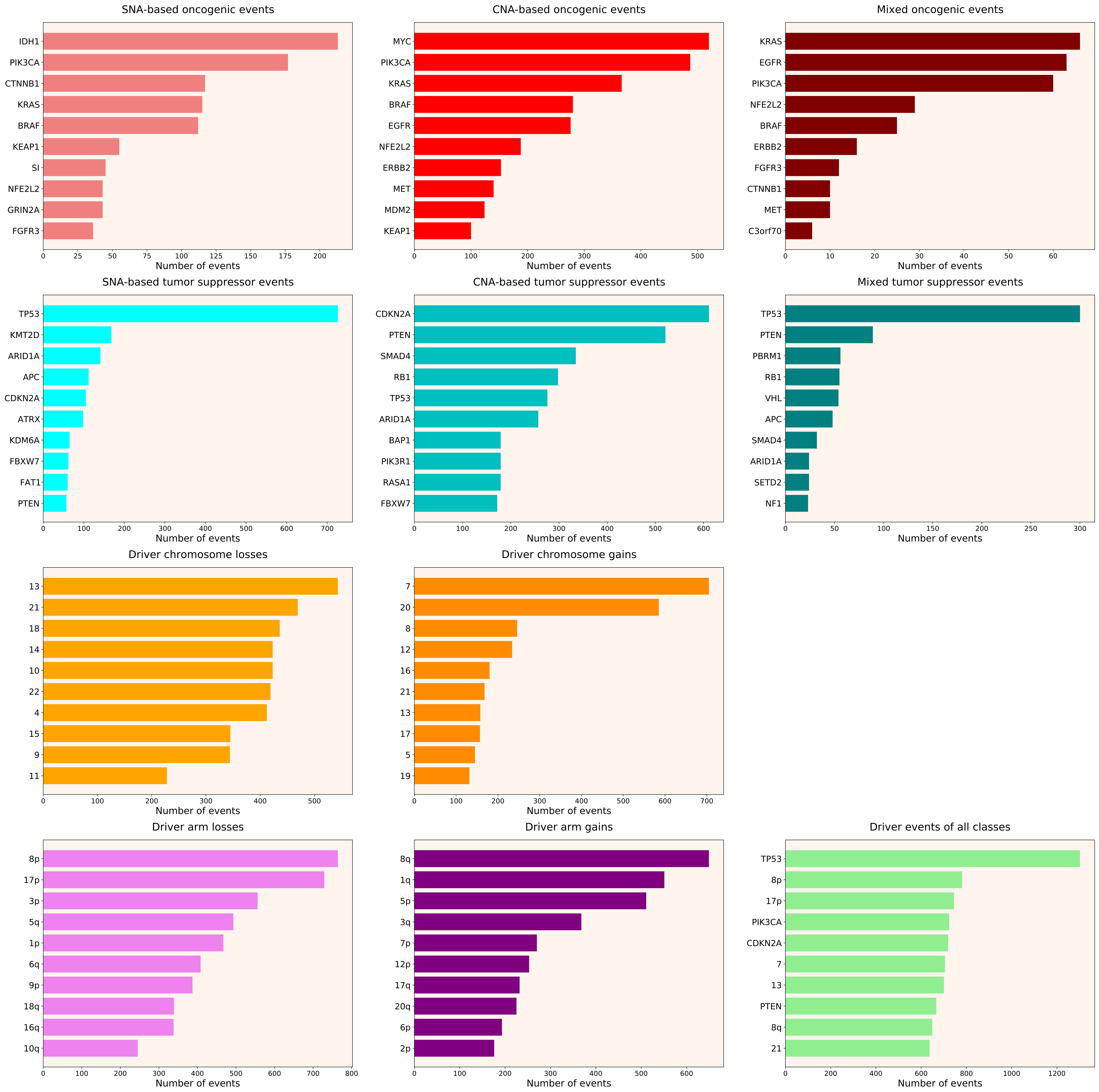
SNA-based oncogenic events
IDH1
PIK3CA
CTNNB1
KRAS
BRAF
KEAP1
SI
NFE2L2
GRIN2A
FGFR3
Number of events
CNA-based oncogenic events
MYC
PIK3CA
KRAS
BRAF
EGFR
NFE2L2
ERBB2
MET
MDM2
KEAP1
Number of events
Mixed oncogenic events
KRAS
EGFR
PIK3CA
NFE2L2
BRAF
ERBB2
FGFR3
CTNNB1
MET
C3orf70
Number of events
SNA-based tumor suppressor events
TP53
KMT2D
ARID1A
APC
CDKN2A
ATRX
KDM6A
FBXW7
FAT1
PTEN
Number of events
CNA-based tumor suppressor events
CDKN2A
PTEN
SMAD4
RB1
TP53
ARID1A
BAP1
PIK3R1
RASA1
FBXW7
Number of events
Mixed tumor suppressor events
TP53
PTEN
PBRM1
RB1
VHL
APC
SMAD4
ARID1A
SETD2
NF1
Number of events
Driver chromosome losses
13
21
18
14
10
22
4
15
9
11
Number of events
Driver chromosome gains
7
20
8
12
16
21
13
17
5
19
Number of events
Driver arm losses
8p
17p
3p
5q
1p
6q
9p
18q
16q
10q
Number of events
Driver arm gains
8q
1q
5p
3q
7p
12p
17q
20q
6p
2p
Number of events
Driver events of all classes
TP53
8p
17p
PIK3CA
CDKN2A
7
13
PTEN
8q
21
Number of events
